# Adipocyte commitment of 3T3-L1 cells is required to support human adenovirus 36 productive replication concurrent with altered lipid and glucose metabolism

**DOI:** 10.3389/fcimb.2022.1016200

**Published:** 2022-09-27

**Authors:** Verónica Márquez, Grisel Ballesteros, Thomas Dobner, Ramón A. González

**Affiliations:** ^1^ Centro de Investigación en Dinámica Celular, Instituto de Investigación en Ciencias Básicas y Aplicadas, Universidad Autónoma del Estado de Morelos, Cuernavaca, Mexico; ^2^ Department of Viral Transformation, Leibniz Institute of Virology, Hamburg, Germany

**Keywords:** human adenovirus 36, obesity, adipocyte commitment, permissive replication, lipid and glucose metabolism

## Abstract

Human adenovirus 36 (HAdV-D36) can cause obesity in animal models, induces an adipogenic effect and increased adipocyte differentiation in cell culture. HAdV-D36 infection alters gene expression and the metabolism of the infected cells resulting in increased glucose internalization and triglyceride accumulation. Although HAdV-D36 prevalence correlates with obesity in humans, whether human preadipocytes may be targeted *in vivo* has not been determined and metabolic reprogramming of preadipocytes has not been explored in the context of the viral replication cycle. HAdV-D36 infection of the mouse fibroblasts, 3T3-L1 cells, which can differentiate into adipocytes, promotes proliferation and differentiation, but replication of the virus in these cells is abortive as indicated by short-lived transient expression of viral mRNA and a progressive loss of viral DNA. Therefore, we have evaluated whether a productive viral replication cycle can be established in the 3T3-L1 preadipocyte model under conditions that drive the cell differentiation process. For this purpose, viral mRNA levels and viral DNA replication were measured by RT-qPCR and qPCR, respectively, and viral progeny production was determined by plaque assay. The lipogenic effect of infection was evaluated with Oil Red O (ORO) staining, and expression of genes that control lipid and glucose metabolism was measured by RT-qPCR. In the context of a viral productive cycle, HAdV-D36 modulated the expression of the adipogenic genes, C/EBPα, C/EBPβ and PPARγ, as well as intracellular lipid accumulation, and the infection was accompanied by altered expression of glucolytic genes. The results show that only adipocyte-committed 3T3-L1 cells are permissive for the expression of early and late viral mRNAs, as well as viral DNA replication and progeny production, supporting productive HAdV-D36 viral replication, indicating that a greater effect on adipogenesis occurs in adipocytes that support productive viral replication.

## Introduction

Obesity, defined as a metabolic disorder caused by excessive caloric intake, is considered a major nutritional burden in both high-income and low-income countries (Romieu et al., 2017), and has become one of the most serious health problems globally. Obesity is a multifactorial disease caused by the interaction of genetic, epigenetic, metabolic, lifestyle, immunologic and environmental factors, and is currently considered a pandemic phenomenon (Chooi et al., 2019; Jaacks et al., 2019). Attempts to explain the increase in obesity in most countries of the world during the last four decades have led to the search for additional factors that may be more adequately adjusted to the characteristics of an epidemiological phenomenon (Dhurandhar, 2001; Blaut and Clavel, 2007; Mitra and Clarke, 2010). The accumulated evidence has shown that some virus infections induce metabolic alterations and have adipogenic effects in different animal species (Dhurandhar et al., 2000; Whigham et al., 2006; Pasarica and Dhurandhar, 2007; Munger et al., 2008; Voss et al., 2015); however, many questions still remain about the role of infectious agents and their contribution to obesity in humans and the current obesity pandemic.

The prevalence of Human Adenovirus 36 (HAdV-D36) has been correlated with obesity in humans (Yamada et al., 2012; Shang et al., 2014; Xu et al., 2015). Moreover, virus infection can cause obesity in animal models (Dhurandhar et al., 2001; Dhurandhar et al., 2002; Atkinson, 2007) and alter lipid metabolism in cellular models (Vangipuram et al., 2004; Pasarica et al., 2008). In animal models HAdV-D36 infection increases adiposity, decreases cholesterol and serum triglycerides, alters the expression of adipogenic genes, increases glucose metabolism and promotes preadipocyte to adipocyte differentiation, as well as cellular proliferation without cell lysis (Pasarica et al., 2008). In cellular models HAdV-D36 infection of rodent preadipocytes increases differentiation and lipid accumulation (Vangipuram et al., 2004), and enhances differentiation of human adipose-derived stem cells (Pasarica et al., 2008). However, detailed studies of the effect of productive virus replication in adipocytes that are permissive to infection is still lacking. Therefore, studies are needed to determine the specific effects of HAdV-D36 infection and replication on adipocytes at different stages of adipocyte differentiation.

The molecular events associated with adipocyte differentiation have been studied in detail in the mouse preadipocytes, 3T3-L1 cells, because these cells can be induced to differentiate in response to dimethyl-ethyl-xanthine (MIX), dexamethasone (DEX), and insulin (MacDougald and Lane, 1995; Yeh et al., 1995), an adipogenic cocktail commonly abbreviated MDI. The adipogenic program follows two sequential phases: the first includes events that favor the commitment of cells to preadipocytes, while the second involves mechanisms that allow mature adipocyte formation (Hershey and Merrick, 2000). Studies of the coordinated activation of Cytosine-Cytosine-Adenosine-Adenosine-Thymidine/Enhancer-binding proteins (C/EBPs) and peroxisome proliferator activated receptor γ (PPARγ) have shown that the induction of C/EBPβ increases expression of C/EBPα, which in turn activates the expression of adipocyte genes and thus stimulates the differentiation process (Tanaka et al., 1997; Gregoire et al., 1998; Salma et al., 2004). PPARγ and C/EBPα are two key regulators of adipogenesis that are necessary for adipocyte differentiation both in cell culture and in animal models, and act in a reciprocal positive feedback loop (Wu et al., 1999; Linhart et al., 2001; Rosen et al., 2002; Farmer, 2005). HAdV-D36 infection increases the expression of C/EBPβ downstream genes, C/EBPα and glycerol-3-phosphate dehydrogenase (GPDH), suggesting that a direct target of HAdV-D36 in the adipocyte differentiation program may be C/EBPβ (Pasarica et al., 2008; Na et al., 2012). C/EBPβ results in activation of C/EBPα and PPARγ at an early stage, which in turn increase adiponectin and fatty acid synthase (FAS) levels for mature adipocyte formation.

**Figure 1:**
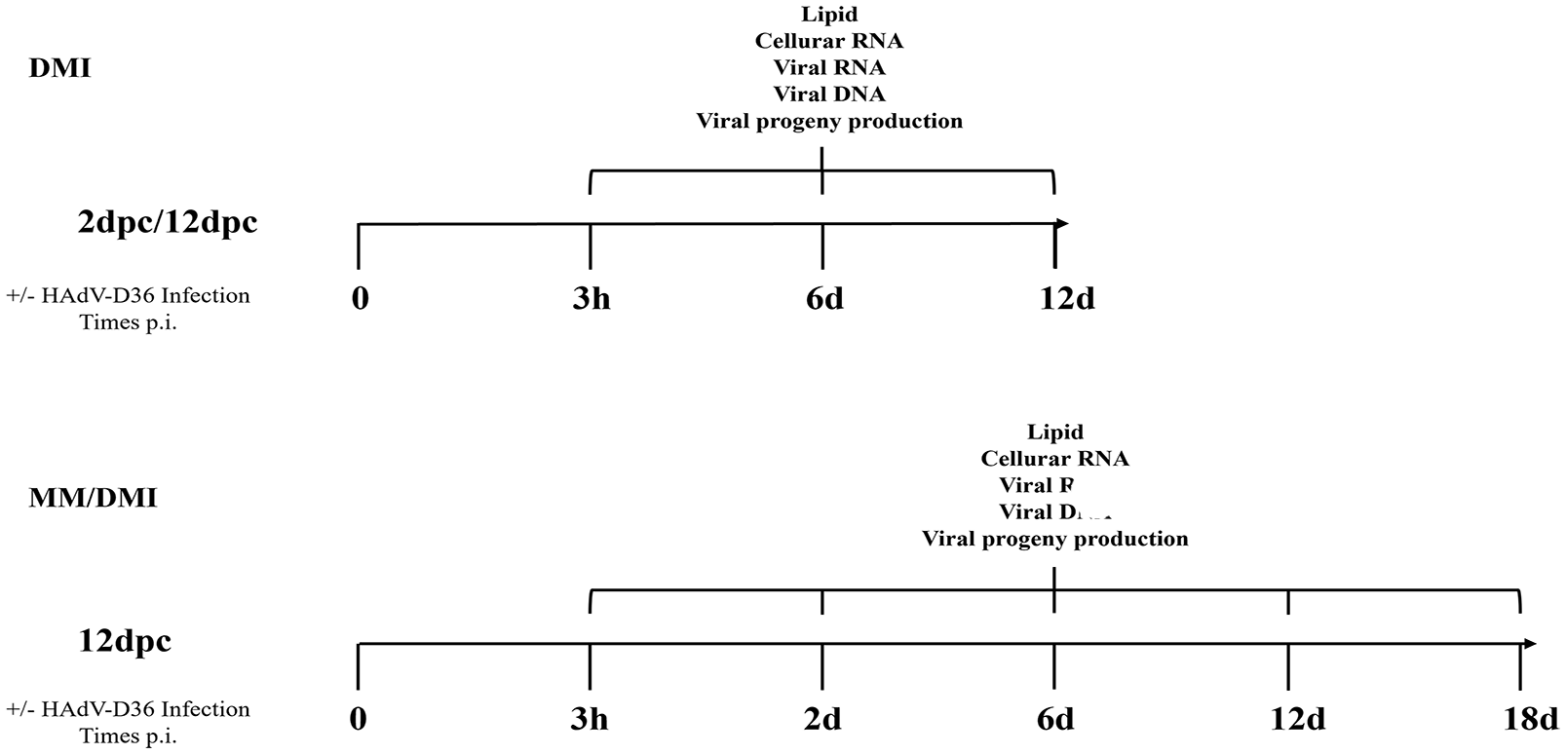
Experiment workflow. 3T3-L1 cells were infected with HAdV-D36 or mock-infected at 2 or 12 days postconfluence (2 dpc or 12 dpc, respectively), in the absence or presence of differentiation factors (MM or MDI, respectively). The cells were harvested at the indicated time-points when measurements of viral and cellular mRNA, viral DNA, viral progeny production and intracellular lipids were performed as described in materials and methods.

Although HAdV-D36 infection of 3T3-L1 promotes cell proliferation and differentiation (Vangipuram et al., 2004; Dubuisson et al., 2011), the viral replication cycle is abortive and cellular metabolism is altered without ensuing cell lysis or cytopathic effect (Rathod et al., 2007). Hence, the effects of HAdV-D36 replication in adipocyte differentiation and lipid metabolism have not yet been fully elucidated. Therefore, to determine under what conditions the cells are permissive for HAdV-D36 replication and to evaluate the adipogenic effect in the context of viral replication, we measured viral early and late mRNA expression, DNA replication and progeny production, as well as genes that are key regulators of the adipogenic cascade, C/EBPα, C/EBPβ and PPARγ, and MYC glycolytic target genes, in 3T3-L1 cells infected with HAdV-D36 at different stages of differentiation (**Figure 1**). The results show that the infection with HAdV-D36 increased the expression of adipogenic genes C/EBPα, C/EBPβ and PPARγ, as well as intracellular lipid accumulation, regardless of the differentiation stage. However, only 3T3-L1 cells that have engaged the differentiation process were permissive for HAdV-D36 replication allowing the expression of early and late viral mRNAs, as well as viral DNA replication and viral progeny production, concurrent with greater metabolic alterations and amplified adipogenic effects.

## Materials and methods

### Cells and viruses

Mouse 3T3-L1 cells were used as a model of preadipocytes. The cells were maintained in monolayer cultures in minimum medium (MM) consisting of Dulbecco’s modified Eagle’s medium (DMEM) with 1.5 g/l NaHCO_3_, supplemented with 10% (vol/vol) bovine serum (BS), 0.1% sodium pyruvate, 100 U/ml penicillin, and 100 µg/ml streptomycin at 37°C in 5% CO_2_. For the differentiation medium (MDI), 0.5 mM dimethyl-ethyl-xanthine, 25 mg/ml dexamethasone, 10 mg/ml insulin were added. The culture medium was replaced with fresh medium every 2 days. Human lung carcinoma A549 cells were maintained in monolayer cultures in DMEM supplemented with 10% (vol/vol) BS, 100 U/ml penicillin and 100 µg/ml streptomycin at 37°C in 5% CO_2_. Human Adenovirus 36 wild type (HAdV-D36) was propagated in A549 cells and virus titers were determined using plaque assays. Briefly, A549 cells were grown in 12-well plates with DMEM/5% BS + 5% Fetal Bovine Serum (FBS). When the cells reached 90% confluence, they were infected and overlaid with agarose (DMEM 2X supplemented with 7.5% NaHCO_3_, 10 mg/ml gentamicin, 1 M MgCl_2_, 2% FBS and 1% agarose). Serial dilutions were used to determine viral titers. When plaque formation was observed (14 days post-infection), 125 µl of neutral red (1% in MilliQ water) was added to the medium and after 4 hours plaques were counted at each of the dilutions to calculate virus titer. The 3T3-L1 cells were infected with HAdV-D36 at a MOI of 5 PFU/cell in all experiments.

### Quantitative PCR and RT-PCR

DNA or RNA was isolated from total lysates of mock-infected (MK) or HAdV-D36-infected cells. 3T3-L1 cells were grown to confluence and 12 days after confluence, were infected with HAdV-D36 at a MOI of 5 PFU/cell. For viral DNA, the cells were collected at 3 hpi, 2 dpi, 6 dpi, 12 dpi and 18 dpi and centrifuged for 5 minutes at 400 g at 4°C; the cell pellet was resuspended in 1 mg/ml Proteinase K (Promega) and 1:200 Tween20 (Promega) and incubated for 1 h at 55°C. After incubation, Proteinase K was inactivated for 10 min at 95°C. The solution was centrifuged for 2 min at 10,000 g and the supernatant was collected. The DNA was precipitated with 1/10 volume of 3M Sodium Acetate and 1 volume of isopropanol at 4°C overnight. The DNA was resuspended in 10 μl of 10 mM Tris pH 7.4 and stored at -20°C until use. For RNA, the cells were collected at 3 hpi, 2 dpi, 6 dpi, 12 dpi and 18 dpi and centrifuged for 5 minutes at 400 g at 4°C; the cell pellet was extracted using TRIzol (Invitrogen) according to the manufacturer’s instructions. The RNA from each sample was quantified using NanoDrop. To analyze RNA, equal volumes (approximately 100 ng) of RNA were reverse transcribed using Revert-Aid reverse transcriptase according to the manufacturer’s instructions (Thermo Scientific) in 20 µl reaction volumes. Viral or cellular genes were quantified using the Power SYBR green PCR master mix kit according to the manufacturer’s instructions (Applied Biosystems). The StepOne system (Applied Biosystems) was used for real-time thermocycling. The cDNA samples were analyzed using the ΔΔCt comparative method. The U1 cellular gene was used as the internal reference and the samples at the earliest time-point were used as the calibrator. All experiments were performed in technical duplicates for two independent experiments.

### Primers

The CLC Sequence Viewer (CLC Bio), Primer Plex (Premier Biosoft), and Primer-BLAST (NCBI) programs were used to design primers specific for the viral and cellular gene sequences of interest. These primers allowed the amplification of a unique product of the expected size, as determined by melt curve analyses. All primers were validated to confirm an amplification efficiency of 100% ± 10%, as calculated by the linear regression obtained from standard curve assays. The primers used to quantify viral and cellular mRNAs are shown in **Table 1**.

**Table 1:** Primers used for qPCR and RT-qPCR.

Target genes	Primer sequence 5’-3’	Amplicon size (bp)
E1A 21k	Fw. CGGCGACCTGGCTGTGATTATG	253
	Rv. TTCAGGTATGGGAGGCAGAGTGG	
L3 Hexón	Fw ATCGCAGTCGCAAATGGCCA	149
	Rv CCCAGGCTGAAGTACGTGTC	
IVa2	Fw. TGGAGACGCGAGGGCGAAG	112
	Rv. ACGTCACCGAGCTCTGGGAC	
HAdV-D36 DNA	Fw. CCGTGTGGTTAAAGAGCAGC	184
	Rv. TTCCACATTCCTCCGCATGG	
HK2	Fw. CGGCCGTGCTACAATAGG	80
	Rv. CTCGGGATCATGTGAGGG	
PFK	Fw GGCGGAGATCACATCAGG Rv GTAATCCCACGCTTCACCAG	88
GAPDH	Fw. CCCACCACACTGAATCTCCC	88
	Rv. TACATGACAAGGTGCGGCTC	
LDHA	Fw. ACGTCAGCATAGCTGTTCCACT	83
	Rv. TGAGATCCGGAATCGGCGG	
PPAR g	Fw GCCTGCGAAAGCCTTTTGGTG	151
	Rv GGCTTCACATTCAGCAAACCTGG	
C/EBP a	Fw AGGAACACGAAGCACGATCAG	141
	Rv CGCACATTCACATTGCACAA	
C/EBP b	Fw CGGACTGCAAGCGGAAGGAGGA	150
	Rv GGCTGGACGACGAGGATGTGGA	

### Intracellular lipid staining

The accumulation of intracellular lipids was determined with the Lipid Oil Red O (ORO) Staining; 3T3-L1 cells were grown in 35 mm culture plates and 12 days after confluence were infected as described above. The cells were fixed for 10 min with 10% formalin; formalin was removed and the cells were gently washed 2X with sterile Milli-Q water. Isopropanol (60%) was added to each well and incubated for 5 min. Isopropanol was removed and Oil Red O Working Solution (0.5% W/V Oil Red/Isopropanol 100%) was added and incubated for 1 h. The cells were washed five times with Milli-Q water and viewed and photographed using bright field microscopy. For ORO quantification, stained cells were treated with 100% isopropanol to elute the staining and absorbance of the eluate was measured at 510 nm.

### Statistical analyses

All data were analyzed with two-way analysis of variance (ANOVA) and multiple t tests using GraphPad 8.0.2 for Microsoft (GraphPad Software, San Diego, CA, USA).

## Results

### 3T3-L1 cells that have initiated adipocyte differentiation are susceptible but not permissive to HAdV-D36 infection

The 3T3-L1 adipocyte model has been used extensively to study the process of cell differentiation and commitment. When the cells reach confluence proliferation is arrested and initial preadipocyte differentiation ensues. By 12 days post-confluence (dpc) the cells commit to differentiation, undergo mitotic clonal expansion and progress to adipocyte maturation (Scott et al., 1982; Wille and Scott, 1982). Cell differentiation and commitment can also be induced by dimethyl-ethyl-xanthine, dexamethasone and insulin (MDI) (Yeh et al., 1995). In both conditions the differentiation process is accompanied at the early phase by increased expression of the C/EBPs adipogenic regulators, which lead to PPARγ expression and lipid accumulation induced by adiponectin and fatty acid synthase (FAS) for mature adipocyte formation at the later phase (Gregoire et al., 1998; Moseti et al., 2016).

**Figure 2:**
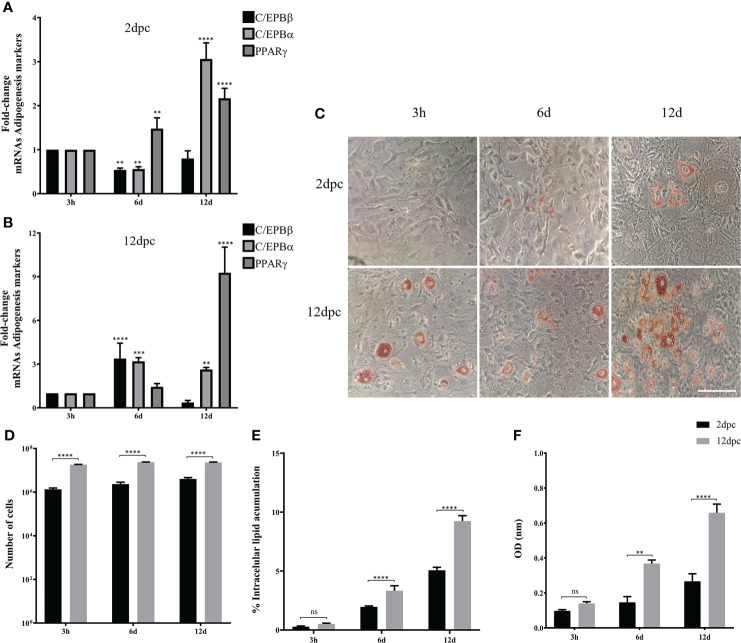
3T3-L1 cells at 12 dpc show characteristics of differentiation committed preadipocytes. Differentiation of 3T3-L1 preadipocytes was induced by incubation postconfluence and addition of MDI. RNA was isolated from 3T3-L1 cells at 2 dpc **(A)** or 12 dpc **(B)**, and expression levels of C/EBPβ, C/EBPα, and PPARγ were analyzed by RT-qPCR. Intracellular lipid accumulation was observed by ORO staining and white field microscopy **(C)**. Scale bar = 100 μm. Cell numbers were measured using a Neubauer chamber **(D)**. Changes in the percentage of cells with lipid accumulation were counted using ORO staining and white field microscopy **(E)**, and the relative quantities of lipid accumulation were determined by elution of ORO staining, measured at 510 nm Optical Density (OD) **(F)**. Primers were designed to hybridize at exon-exon junctions to measure mature mRNA. All values represent the mean of two independent experiments, measured in technical duplicates. Data are expressed as the mean and error bars represent standard deviations. Significant differences from each time-point relative to 3h **(A, B)** and between Mock- and HAdV-D36-infected cells at each time-point **(D-F)** are indicated by **p < 0,005. ***p < 0,0005. ****p < 0,00001. ns, not significant.

To evaluate if the phase of cell differentiation determines whether the 3T3-L1 cells are permissive for HAdV-D36 replication, and analyze the effect of productive viral replication on adipogenesis, we decided to compare the effect of HAdV-D36 infection in 3T3-L1 cells at 2 and 12 dpc, in the presence of MDI. Initially the expression levels of C/EPBβ, C/EPBα, PPARγ mRNAs and lipid accumulation were measured comparing uninfected 3T3-L1 cells in medium supplemented with MDI at 2 and 12 dpc. The cells were then harvested at 3 h, 6 d and 12 d after MDI addition (**Figure 2**), as described in the Materials and Methods section. The cellular mRNAs were measured by quantitative reverse transcription PCR (RT-qPCR) with primers targeting mature mRNA sequences, and lipid accumulation was measured with the ORO reagent. As expected, the cell number increased approximately ten-fold from 2 dpc to 12 dpc in the presence of MDI, but only approximately 2-fold every 6 days when cells at 2 dpc were stimulated with MDI, and increased only by approximately 30% between 2 and 6 days when MDI was added to cells at 12 dpc (**Figure 2D** and **Table 2**). Also as expected, both the percentage of cells (**Figure 2E**) and the total lipid accumulation per cell (**Figure 2F**) was significantly higher in the cells supplemented with MDI at 12 dpc. The levels of C/EBPβ and C/EBPδ have been shown to increase transiently during the early phase of differentiation and to decrease at the later stage, when C/EPBα and consequently PPARγ accumulate leading to synthesis and storage of fatty acids (MacDougald and Lane, 1995). Significant differences in the patterns of expression of CEBPs and PPARγ were observed between the cells at 2 dpc and 12 dpc (**Figures 2A, B**). The cells at 12 dpc displayed 2 to 4-fold higher levels and a pattern of C/EPBβ, C/EPBα, PPARγ mRNAs that would be expected for committed adipocytes at 12 d after MDI addition to the medium (**Figure 2B**), where a transient increase of C/EPBβ and C/EPBα was followed by higher levels of PPARγ.

**Table 2:** Number of 3T3-L1 cells at different time-points at 2 and 12 dpc in MDI.

Time	Number of cells	
	2dpc	12dpc
3h	1.36 X 10^6^	1.82 X 10^7^
6d	2.35 X 10^6^	2.36 X 10^7^
12d	4.02 X 10^6^	2.30 X 10^7^

**Figure 3:**
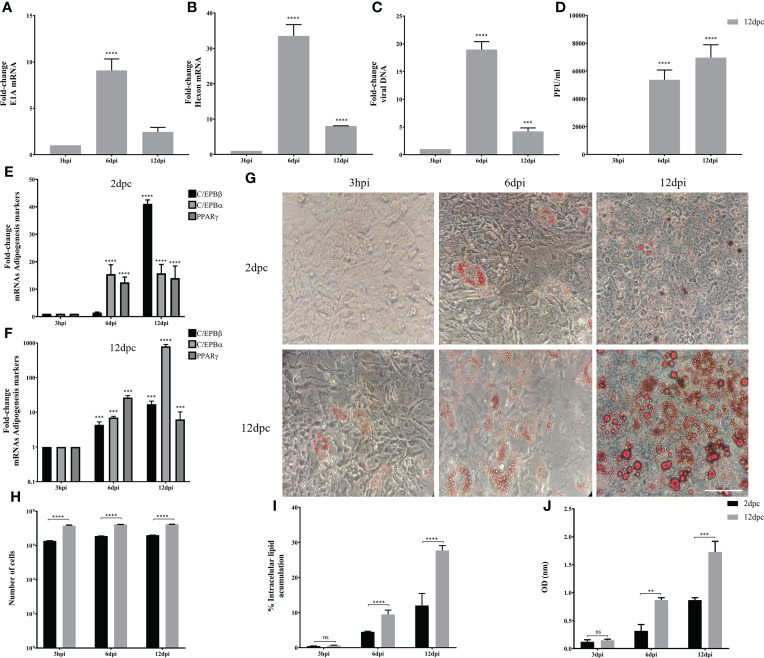
3T3-L1 cells at 12 dpc, but not at 2 dpc, are permissive for HAdV-D36 replication. 3T3-L1 cells, 2dpc or 12dpc, were infected with HAdV-D36 in the presence of MDI. Viral mRNA expression levels of E1A **(A)**, and Hexon **(B)** were measured by RT-qPCR. DNA was isolated and analyzed by qPCR **(C)** and viral progeny production was determined by plaque assay **(D)**. The expression levels of C/EBPβ, C/EBPα and PPARγ was measured by RT-qPCR **(E, F)**. Intracellular lipid accumulation was observed by ORO staining and white field microscopy **(G)**. Scale bar = 100 μm. Cell numbers were measured using a Neubauer chamber **(H)**. Change in the percentage of cells with lipid accumulation in the presence of MDI were counted using ORO staining and white field microscopy **(I)**, the relative quantities of lipid accumulation were determined by elution of ORO staining, measured at 510 nm Optical Density (OD) **(J)**. Primers were designed to hybridize at exon-exon junctions to measure mature mRNA and to hybridize at sequences that correspond to intron-exon junctions to measure DNA. All values represent the mean of two independent experiments, measured in technical duplicates. Data are expressed as the mean and error bars represent standard deviations. Significant differences from each time-point relative to 3h **(A–F)** and between 2 and 12 dpc at each time-point **(H-J)** are indicated by **p < 0,005. ***p < 0,0005. ****p < 0,00001. ns, not significant.

Using the same experimental set-up, we then evaluated the conditions in which 3T3-L1 cells are permissive for HAdV-D36 replication. The 3T3-L1 cells supplemented with MDI at 2 and 12 dpc were infected at a MOI of 5 PFU/cell. DNA and RNA were isolated and viral early and late mRNA expression levels, as well as viral DNA and progeny production were determined at 3 hours, 6 days and 12 days post infection (hpi or dpi). The viral early, E1A, and viral late, hexon, mRNAs were chosen because E1A expression is required to induce expression of all viral genes and the hexon gene is only expressed after viral DNA replication initiates (Berk, 2013). Very low and transient levels of viral mRNAs were measured in cells infected at 2 dpc, and no viral DNA replication or progeny production were detected (data not show), in agreement with previous results (Rathod et al., 2007). In contrast, steady state levels of both E1A and hexon mRNA increased when the cells were infected at 12 dpc. Interestingly, E1A and hexon mRNA, and viral DNA peaked at 6 dpi, and although viral progeny production accumulated until 12 dpi it did not significantly increase after 6 dpi (**Figures 3A-D**). These results indicate that in the presence of MDI at 2 dpc the 3T3-L1 cells are susceptible to infection, but only cells at 12 dpc are permissive for productive viral replication. The patterns of C/EBPβ, C/EBPα and PPARγ differed markedly between the cells infected at 2 and 12 dpc (**Figures 3E, F**). High C/EBPβ levels were detected at 12 dpi in the cells infected at 2 dpc, but were not accompanied by higher PPARγ. In contrast, higher levels of PPARγ were observed in the cells infected at 12 dpc at 6 dpi, but accumulation of C/EBPα was higher at 12 dpi, an expression pattern that was different from the uninfected cells (compare **Figures 2A, B** with **Figures 3E, F**). Both the percentage of cells and the total lipid accumulation increased sharply in the cells infected at 12 dpc, while the lipid increase in cells infected at 2 dpc was similar to that of uninfected cells (compare **Figures 2C–F** to **Figures 3G–J**). As mentioned above, after differentiation is induced the preadipocytes undergo postconfluent mitosis with at least one round of DNA replication and cell division, and subsequent growth arrest (Scott et al., 1982; Wille and Scott, 1982). In the infected cells the number of cells at 2 dpc (**Figure 3H** black bars and **Table 3**) doubled by 6 dpi and continued to proliferate. In contrast, cells at 12 dpc increased only by 20%, and there was no increase by 12 dpi (**Figure 3H** gray bars and **Table 3**).

**Table 3:** Number of 3T3-L1 cells at different time-points. HAdV-36-infected at 2 and 12 days dpc in MDI.

Time post infection	Number of cells	
	2dpc	12dpc
3h	1.86 X 10^6^	1.42 X 10^7^
6d	3.52 X 10^6^	1.70 X 10^7^
12d	4.00 X 10^6^	1.73 X 10^7^

### 3T3-L1 adipocyte commitment is required to support HAdV-D36 productive replication

**Figure 4:**
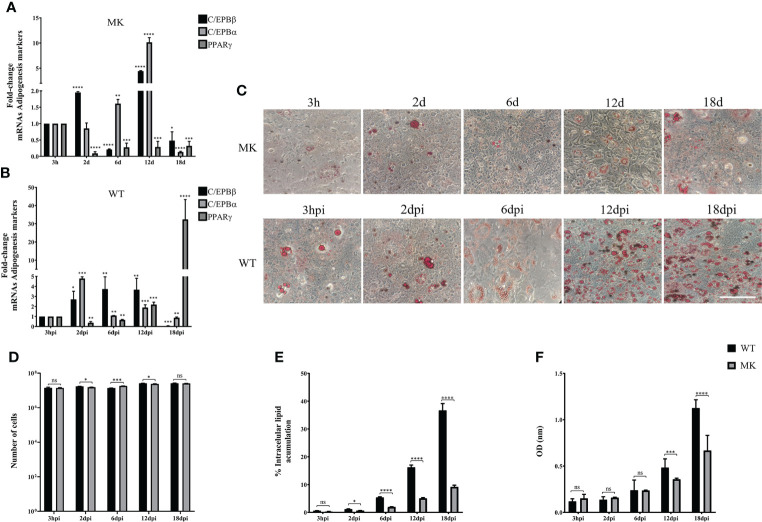
HAdV-D36 infection increases adipogenesis in 3T3-L1 cells. 3T3-L1 cells at 12 dpc were infected with HAdV-D36 without adipocyte differentiation inducers (MM) and the expression levels of C/EBPβ, C/EBPα and PPARγ were analyzed by RT-qPCR **(A, B)**. Intracellular lipid accumulation was observed by ORO staining and white field microscopy **(C)**. Scale bar = 100 μm. Cell numbers were measured using a Neubauer chamber **(D)**. Change in the percentage of cells with lipid accumulation in the absence of MDI were counted using ORO staining and white field microscopy **(E)**, the relative quantities of lipid accumulation were determined by elution of ORO staining, measured at 510 nm Optical Density (OD) **(F)**. Primers were designed to hybridize at exon-exon junctions to measure mature mRNA. All values represent the mean of two independent experiments, measured in technical duplicates. Data are expressed as the mean and error bars represent standard deviations. Significant differences from each time-point relative to 3h **(A, B)** and between Mock- and HAdV-D36-infected cells at each time-point **(D-F)** are indicated by *p < 0,05. **p < 0,005. ***p < 0,0005. ****p < 0,00001. ns, not significant.

**Figure 5:**
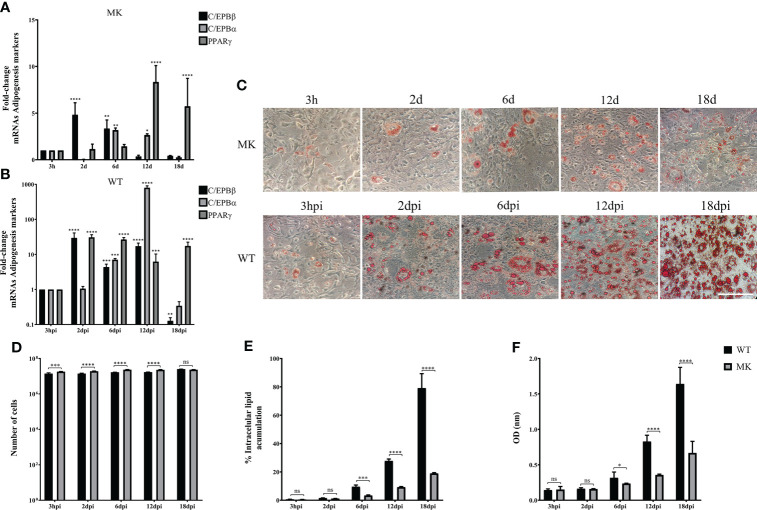
The effect of HAdV-D36 on 3T3-L1 adipogenesis is enhanced by adipocyte differentiation inducers. The effect of infection on 12 dpc 3T3-L1 cells cultured in MDI was evaluated. 12 dpc 3T3-L1 cells were infected with HAdV-D36 in the presence of adipocyte differentiation inducers (MDI) and the expression levels of C/EBPβ, C/EBPα and PPARγ were analyzed by RT-qPCR **(A, B)**. Intracellular lipid accumulation was observed by ORO staining and white field microscopy **(C)**. Scale bar = 100 μm. Cell numbers were measured using a Neubauer chamber **(D)**. Change in the percentage of cells with lipid accumulation in the presence of MDI were counted using ORO staining and white field microscopy **(E)** the relative quantities of lipid accumulation were determined by elution of ORO staining, measured at 510 nm Optical Density (OD) **(F)**. Primers were designed to hybridize at exon-exon junctions to measure mRNA. All values represent the mean of two independent experiments, measured in technical duplicates. Data are expressed as the mean and error bars represent standard deviations. Significant differences from each time-point relative to 3h **(A, B)** and between Mock- and HAdV-D36-infected cells at each time-point **(D-F)** are indicated by *p < 0,05. **p < 0,005. ***p < 0,0005. ****p < 0,00001. ns, not significant.

Taken together, the results described above suggest that adipocyte commitment may be required to support HAdV-D36 productive viral replication, and that the effect of HAdV-D36 infection on adipogenesis may be greater when cells are committed to adipocytes. Therefore, we decided to compare C/EBPβ, C/EBPα and PPARγ mRNAs, and lipid levels as above, in both mock-infected (MK) and HAdV-D36-infected 3T3-L1 cells at 12 dpc, in the absence (**Figure 4**) or in the presence (**Figure 5**) of MDI. The cells were harvested at 3 h, 2 d, 6 d, 12 d and 18 d (3 hpi, 2 dpi, 6 dpi, 12 dpi and 18 dpi for infected cells). The 2 and 18 d time-points were included in these experiments in an attempt to gain better insight into the initial effect of MDI, and because PPARγ mRNA levels were lower than those of C/EBPα mRNA at 12 dpi in cells infected both at 2 and 12 dpc, suggesting the possibility that the cells have not reached a mature adipocyte phase by 12 days. The data obtained with these experiments are shown in **Figures 4**, **5** and **Table 4**, **5**, where complex patterns and marked differences in the mRNA levels of C/EBPβ, C/EBPα and PPARγ were observed between all conditions tested. However, only in the infected cells in the presence of MDI, 10- to 500-fold increments in the levels of the adipogenesis markers were observed (compare **Figures 4A, B** with **Figures 5A, B**). In the absence of MDI in the MK-infected cells the levels of C/EBPβ and C/EBPα mRNA increased reaching a maximum level at 12 d, but the levels of PPARγ mRNA decreased by 2 d and increased only slightly by 6 d remaining at a similar level up to the 18 d time-point (**Figure 4A**), when only about 10% of the cells displayed lipid accumulation (**Figure 4E**). The infection with HAdV-D36 induced approximately 2-fold and 5-fold rise in C/EBPβ and C/EBPα mRNA, respectively, by 2 dpi. PPARγ mRNA levels increased 2-fold by 12 dpi, and 30-fold by 18 dpi (**Figure 4B**), when approximately 40% of the cells accumulated lipids (**Figures 4C, E**). As expected, the presence of MDI was sufficient to induce a significant rise in the levels of C/EBPβ and C/EBPα, and approximately 7-fold increase in PPARγ mRNA levels by 12 d (**Figure 5A**), with close to 20% of the cells displaying lipid accumulation (**Figures 5C, E**). However, a much sharper increase in C/EBPβ, C/EBPα and PPARγ mRNAs was observed in HAdV-D36-infected cells. Nearly 80% of the cells accumulated more than 2-fold higher levels of total lipids (**Figures 5B, C, E, F**), confirming that the most pronounced adipogenic effect was induced in cells infected at 12 dpc in the presence of MDI.

**Table 4:** Number of 3T3-L1 cells at different time-points. HAdV-36-infected vs MK-infected at 12 dpc in MM.

Time post infection	Number of cells	
	WT	MK
3h	1.42 X 10^7^	1.39 X 10^7^
2d	1.73 X 10^7^	1.53 X 10^7^
6d	1.39 X 10^7^	1.81 X 10^7^
12d	2.61 X 10^7^	2.40 X 10^7^
18d	2.62 X 10^7^	2.55 X 10^7^

**Table 5:** Number of 3T3-L1 cells at different time-points. HAdV-36-infected vs MK-infected at 12 dpc in MDI.

Time post infection	Number of cells	
	WT	MK
3h	1.42 X 10^7^	1.82 X 10^7^
2d	1.42 X 10^7^	1.94 X 10^7^
6d	1.70 X 10^7^	2.36 X 10^7^
12d	1.73 X 10^7^	2.30 X 10^7^
18d	2.55 X 10^7^	2.35 X 10^7^

**Figure 6:**
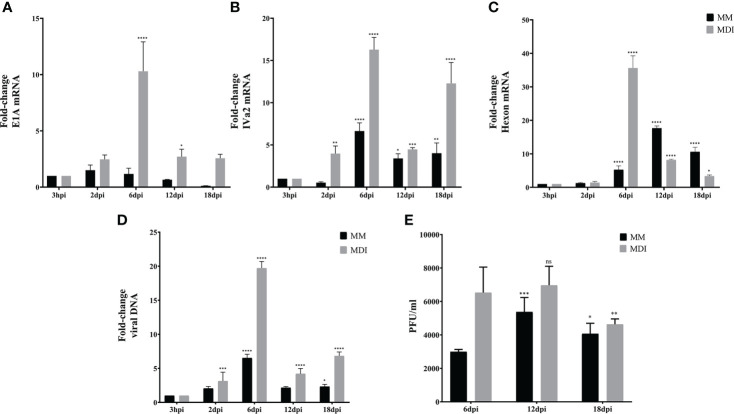
Adipocyte commitment of 3T3-L1 cells is required to support productive replication of HAdV-D36. 3T3 cells treated with or without adipocyte differentiation inducers (MDI or MM) were infected 12dpc, and RNA and DNA were purified as described in materials and methods. Expression levels of E1A **(A)**, IVa2 **(B)** and Hexon **(C)** were measured by RT-qPCR and DNA by qPCR **(D)**. Viral progeny production was determined by plaque assay **(E)**. Primers were designed to hybridize at exon-exon junctions to measure mature mRNA and at sequences that correspond to intron-exon junctions to measure DNA. All values represent the mean of two independent experiments, measured in technical duplicates. Data are expressed as the mean and error bars represent standard deviations. Significant differences from each time-point relative to 3h **(A-D)** and relative to 6 dpi **(E)** are indicated by *p < 0,05. **p < 0,005. ***p < 0,0005. ****p < 0,00001. ns, not significant.

As described in the experiments in **Figure 3**, as expected (Rathod et al., 2007), barely detectable and transient levels of E1A and hexon mRNA, and no viral DNA replication or virus production were observed in the 3T3-L1 cells infected at 2 dpc in the presence of MDI. Therefore, to evaluate the effect of the phase of adipocyte differentiation on HAdV-D36 productive replication, we then measured viral early (E1A), intermediate (IVa2), and late (hexon) mRNAs, viral DNA replication, and viral progeny production in 3T3-L1 cells infected at 12 dpc, with or without MDI. The IVa2 mRNA was included in these experiments because the IVa2 protein is required for the transition of the early to the late phase of adenovirus replication and the activation of the viral late genes, which depends on active viral DNA replication during productive virus replication. As before, the cells were harvested at 3 hpi, and 2, 6, 12 and 18 dpi. Interestingly, although transiently the cells infected at 12 dpc, both in the absence or presence of MDI, were permissive for HAdV-D36 replication (**Figure 6**). In the absence of MDI, lower levels of the viral mRNA and DNA were produced (**Figures 6A–D**, black bars); however, they were sufficient to sustain production of viral progeny, which could be initially detected by 6 dpi and peaked by 12 dpi (**Figure 6E**). The accumulation of E1A mRNA was very low and transient, and was followed by increased IVa2 expression, which peaked at 6 dpi, and later by hexon mRNA accumulation, which was delayed and reached a maximum level at 12 dpi. In the presence of MDI 2- to 5-fold higher levels of the viral mRNAs were measured, which reached a maximum level at 6 dpi, with a pattern of accumulation that more closely paralleled that of viral DNA (**Figure 6D**, grey bars). These results confirm that viral gene expression and DNA replication peak when higher levels of both C/EBPα and PPARγ mRNAs are reached (**Figure 5B**), and that adipocyte committed 3T3-L1 cells support HAdV-D36 productive viral replication, with a greater effect on increased adipogenesis.

### HAdV-D36 infection promotes glucose metabolism through MYC target genes in adipocyte committed 3T3-L1 cells

**Figure 7:**
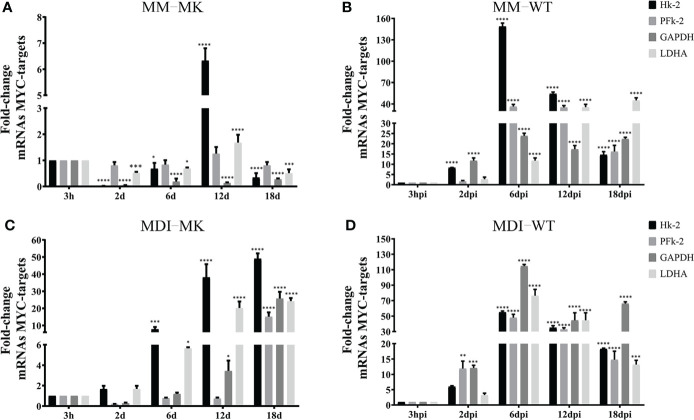
HAdV-D36 infection of adipocyte committed cells promotes the expression of MYC- glycolytic target genes. To determine the effect of infection on MYC-target genes, 3T3-L1 cells at 12dpc were HAdV-D36-infected **(B, D)** or mock-infected **(A, C)**, without adipocyte differentiation inducers (MM) **(A, B)** or with (MDI) **(C, D)**. RNA was isolated at the indicated time-points and the expression levels of hexokinase (Hk), phosphofructokinase (PFk), glyceraldehyde-3-phosphate dehydrogenase (GAPDH) and Lactate dehydrogenase (LDHA) were measured by RT-qPCR. All values represent the mean of two independent experiments, measured in technical duplicates. Data are expressed as the mean and error bars represent standard deviations. Significant differences from each time-point relative to 3h are indicated by *p < 0,05. **p < 0,005. ***p < 0,0005. ****p < 0,00001. ns, not significant.

The metabolic changes induced by HAdV that contribute to the adipogenic effect of the infection are accompanied with increased Myc transcriptional activation of glycolytic genes (Thai et al., 2014) (Prusinkiewicz et al., 2020). Therefore, we wished to determine whether the activation of Myc target genes for glucose metabolism require conditions in the infected cell that support efficient viral replication. The mRNAs of the hexokinase (Hk), phosphofructokinase (PFk), glyceraldehyde-3-phosphate dehydrogenase (GAPDH) and Lactate dehydrogenase (LDHA) Myc target genes were measured in 3T3-L1 cells that were Mock-infected or infected with HAdV-D36 at 12 dpc, in the absence (MM) or presence (MDI) of adipocyte differentiation inducers, at the same time-points as the previous experiments (**Figure 7**). Except for an unexpected 6-fold rise in the level of the Hk-2 mRNA at 12d, relatively small variations in the levels of mRNA were observed in Mock-infected cells in the absence of MDI through 30 days of cell culture (12 dpc + 18 d), which may be due to the addition of fresh medium to the contact-arrested cells every 48 hrs (**Figure 7A**). In contrast, the sole presence of MDI in the Mock-infected cells induced a rapid and progressive 5- to 50-fold rise in the levels of mRNA (**Figures 7A, C**), as would be expected to support the bioenergetic and biosynthetic demands of adipocyte differentiation and lipid accumulation (**Figure 2**). The adipocyte differentiation inducers showed a clear effect on the increase of Hk-2 and LDHA by 6 d, and all four mRNA increased by at least 10-fold by 18 d. The effect of infection on the rise of the mRNA levels of all four glycolytic genes was higher than MDI, when already by 2 dpi approximately 10-fold higher levels of Hk-2 and PFk-2 were measured, and all four mRNA increased between 15- and 150-fold by 6 dpi. Interestingly, an even more pronounced effect was induced in HAdV-D36-infected cells when in the presence of MDI. A general 50- to 100-fold rise in the levels of Hk-2, PFk, GAPDH and LDHA reached maximum levels that were concurrent with both viral gene expression and DNA replication (**Figures 6A-D**), and the highest levels of C/EBPα and PPARγ (**Figure 5B**). These data suggest that the adipocyte commitment that supports HAdV-D36 productive replication correlates with Myc-dependent activation of glucose metabolism.

## Discussion

Extensive evidence has been obtained of the effect of HAdV-D36 on adipogenesis in animal models (Dhurandhar et al., 2000; Dhurandhar et al., 2001; Dhurandhar et al., 2002, Pasarica et al., 2006), and it has been established that the infection induces preadipocyte differentiation and lipid accumulation in both the 3T3-L1 preadipocyte model (Vangipuram et al., 2004), and in human adipose-derived stem/stromal cells (Pasarica et al., 2008). However, studies of the differentiation phase in which the cells are susceptible and permissive to HAdV-D36 replication leading to viral progeny production are lacking, as is the analysis of the effect of virus replication on adipogenesis. Our results show that 3T3-L1 cells at the initial phase of adipocyte differentiation are susceptible to infection with HAdV-D36, but the expression of viral early genes that is required for viral DNA replication and ensuing late gene expression, was sustained only in cells that were committed to differentiation (**Figures 3A–D**). In agreement with previous reports, either the effect of the penton and penton-base proteins during initial stages of infection (McIntosh et al., 1971) or the low and transient expression of viral early genes (Rathod et al., 2007) may have been sufficient to induce increased C/EBPβ, C/EBPα and PPARγ mRNAs and lipid accumulation (**Figures 3E–G**), but only to levels that were comparable to or slightly higher than those induced by MDI in the MK-infected cells (compare **Figures 3E, G, I, J** with **Figures 5A, C, E, F**). In stark contrast, HAdV-D36 infection of committed adipocytes resulted in several-fold higher expression of adipogenic markers and lipid levels (compare **Figure 4** and **Figure 5**). Interestingly, although 3- to 10-fold higher levels of viral mRNA or viral DNA were produced in cells infected at 12 dpc in the presence of DMI, the differentiation phase of the 3T3-L1 cells at this stage was sufficient to support comparable levels of viral progeny in the absence of the differentiation inducers (**Figures 4**–**6**). These findings indicate that at this stage of differentiation 3T3-L1 cells support productive viral replication and display the most pronounced alteration in lipid metabolism.

It is well established that HAdVs alter the metabolism of the infected cell through a variety of mechanisms that include glucose, glutamine and lipid metabolism to meet the bioenergetic and biosynthetic demands of viral macromolecular synthesis and progeny production (Prusinkiewicz and Mymryk, 2019). HAdV productive replication in permissive cells follows a highly complex viral gene expression program that induces changes in cellular gene expression, signaling pathways and metabolism. Such changes in the infected cell are dynamic and differ as the viral replication cycle progresses (Thai et al., 2015; Carinhas et al., 2017; Valdés et al., 2018). In our experiments, HAdV-D36 viral gene expression, DNA replication and progeny reached maximum levels between 6 and 12 dpi and declined by 18 dpi (**Figure 6**), suggesting that as the adipocytes mature they become less permissive. Therefore, to gain further insight into the effect of HAdV-D36 infection of adipocytes it will be interesting to determine in further detail in what phase of differentiation the cells are most permissive and support the highest level of virus reproduction and propagation.

The activities of various HAdV gene products that reprogram every step of cellular gene expression, from chromatin remodeling and transcription, to posttranscriptional processing, RNA export and mRNA translation, have been studied extensively (Babich and Nevins, 1981; Babiss et al., 1985; Pilder et al., 1986; Hardy et al., 1989; Huang and Hearing, 1989; Chang and Shenk, 1990; Hayes et al., 1990; Mathews, 1990; Nordqvist and Akusjärvi, 1990; Bridge et al., 1991; Sandler and Ketner, 1991; Shenk and Flint, 1991; Nordqvist et al., 1994; Tribouley et al., 1994; Lutz and Kedinger, 1996; Lutz et al., 1997; Hershey et al., 2019; Cuesta et al., 2001; Hidalgo et al., 2016; Hidalgo et al., 2022), but their role on the adipogenic effect of HAdV has not been elucidated. To date the only HAdV protein that has been directly linked to the metabolic alterations that result from HAdV infection is the E4Orf1 protein, which interacts with a group of cellular proteins through a PDZ binding domain motif, resulting in activation of PI3K and AKT (Frese et al., 2003; Javier and Rice, 2011). In the case of HAdV-D36, E4Orf1 displays adipogenic effects that depend on increased expression of glucose receptors and glucose uptake (Rogers et al., 2008b; Wang et al., 2008; Krishnapuram et al., 2011; Mostofinejad et al., 2021); increased fatty acid synthase and conversion of glucose to fatty acids (Vangipuram et al., 2007, Kusminski et al., 2015); and the activation of CBEPs and PPAR-γ that results in adipocyte differentiation (Vangipuram et al., 2004; Farmer, 2005; Pasarica et al., 2006; Jiao et al., 2017; Afruza et al., 2020). The E4Orf1 protein is sufficient to induce the above adipogenic effects when expressed in transfected cells or through lentiviral vectors (Rogers et al., 2008a; Dhurandhar et al., 2011; Dubuisson et al., 2011), but its adipogenic effect in the context of productive viral replication is incompletely understood. Furthermore, in addition to the well characterized effect of E4Orf1 on metabolic pathways, the E1A viral proteins can also alter metabolism (Tessier et al., 2021). The metabolic effect of E1A is most likely predominantly dependent on the protein’s interactions with retinoblastoma (pRB), which results in activation of S phase E2F transcription factors, and with the p300/CBP lysine acetylases (Ferrari et al., 2014), leading to progression of the cell cycle and a sustained proliferative state (Berk, 2013). E1A interacts with Myc-containing complexes and may result in regulation of Myc-target genes impacting energy metabolism through activation of genes involved in glycolysis, glutamine metabolism and mitochondrial biogenesis (Shim et al., 1997; Osthus et al., 2000; Li et al., 2005; Gao et al., 2009; Voss et al., 2015). Interestingly, the E4Orf1 protein produced by HAdV-D9 and HAdV-C5 binds Myc leading to increased transcription of Myc target genes and greater expression of glycolytic enzymes (Thai et al., 2014; Kong et al., 2015). Our results now show that HAdV-D36 infection of 3T3-L1 cells also results in increased levels of glycolytic Myc-target genes displaying a clear effect on Hk-2 and PFk, in agreement with previous findings (Thai et al., 2014; Kong et al., 2015) (**Figure 7**), suggesting that the HAdV-D36 E4Orf1 may stimulate glucose metabolism through Myc, in addition to its effect through PI3K.

Although the correlation of HAdV-D36 with obesity in humans has been reported in many studies (Xu et al., 2015), several questions remain, including whether obese subjects are more susceptible to the infection than non-obese subjects or if the infection causes or increases obesity. A few studies have shown that HAdV-D36 DNA is present in the adipose tissue of some individuals (Goossens et al., 2011). However, evidence of virus replication in human adipose tissue is still lacking. Our findings reveal that the state of differentiation of the 3T3-L1 adipocytes determines whether the cells are permissive for viral replication, and that the greater effects on Myc-glycolytic target genes, C/EBPβ, C/EBPα, PPARγ expression, and lipid accumulation occur in adipocytes that support productive viral replication. These findings therefore warrant further detailed studies of the impact of the HAdV-D36 replication on adipogenesis, both in the 3T3-L1 model adipocyte, and in human preadipocytes. In particular, transcriptomic analysis of HAdV-D36 infected cells at different stages of human adipocyte differentiation should lead to identification of viral and cellular genes implicated in adipogenesis, and produce functional insights of HAdV-D36 association with obesity.

## Data availability statement

The original contributions presented in the study are included in the article/supplementary material. Further inquiries can be directed to the corresponding author.

## Author contributions

VM and GB: investigation, methodology, formal analysis, software, writing - original draft and editing. TD and RG: funding acquisition, investigation, methodology, formal analysis, supervision, writing - review and editing. All authors contributed to the article and approved the submitted version.

## Funding

This work was supported by grants from the Consejo Nacional de Ciencia y Tecnología - Mexico CONACyT-BMBF (267746), SRE-CONACyT (280365), the Bundesministerium für Bildung und Forschung - Germany (01DN16031), the Research Group Linkage Program of the Alexander von Humboldt Foundation, and Verónica Márquez received a fellowship from CONACyT (818465).

## Acknowledgments

We thank Gabriela Rosas Salgado (School of Medicine, UAEM) for providing the 3T3-L1 cells and Fernando Esquivel (School of Medicine, UAEM) for critical reading of the manuscript. We thank Paloma Hidalgo (CIDC UAEM; presently Leibniz Institute of Virology) for qPRC supervision.

## Conflict of interest

The authors declare that the research was conducted in the absence of any commercial or financial relationships that could be construed as a potential conflict of interest.

## Publisher’s note

All claims expressed in this article are solely those of the authors and do not necessarily represent those of their affiliated organizations, or those of the publisher, the editors and the reviewers. Any product that may be evaluated in this article, or claim that may be made by its manufacturer, is not guaranteed or endorsed by the publisher.
